# Pulse Wave Velocity in Atherosclerosis

**DOI:** 10.3389/fcvm.2019.00041

**Published:** 2019-04-09

**Authors:** Hack-Lyoung Kim, Sang-Hyun Kim

**Affiliations:** Division of Cardiology, Department of Internal Medicine, Boramae Medical Center, Seoul National University College of Medicine, Seoul, South Korea

**Keywords:** arterial stiffness, atherosclerosis, cardiovascular disease, pulse wave velocity, risk stratification

## Abstract

Early detection of subclinical atherosclerosis is important to reduce patients' cardiovascular risk. However, current diagnostic strategy focusing on traditional risk factors or using risk scoring is not satisfactory. Non-invasive imaging tools also have limitations such as cost, time, radiation hazard, renal toxicity, and requirement for specialized techniques or instruments. There is a close interaction between arterial stiffness and atherosclerosis. Increased luminal pressure and shear stress by arterial stiffening causes endothelial dysfunction, accelerates the formation of atheroma, and stimulates excessive collagen production and deposition in the arterial wall, leading to the progression of atherosclerosis. Pulse wave velocity (PWV), the most widely used measure of arterial stiffness, has emerged as a useful tool for the diagnosis and risk stratification of cardiovascular disease (CVD). The measurement of PWV is simple, non-invasive, and reproducible. There have been many clinical studies and meta-analyses showing the association between PWV and coronary/cerebral/carotid atherosclerosis. More importantly, longitudinal studies have shown that PWV is a significant risk factor for future CVD independent of well-known cardiovascular risk factors. The measurement of PWV may be a useful tool to select subjects at high risk of developing subclinical atherosclerosis or CVD especially in mass screening.

## Introduction

Cardiovascular disease (CVD) is the leading cause of morbidity and mortality globally (1). In the majority of cases, the underlying cause of CVD is atherosclerosis (2). Atherosclerosis is a progressive disease characterized by the accumulation of lipids, inflammatory cells, and fibrous elements in the wall of large arteries causing progressive luminal narrowing of the arteries (3). Narrowed arteries limit the flow of oxygen-rich blood to vital organs such as the brain and the heart, leading to myocardial and cerebral ischemia. Sometimes, acute occlusion due to the formation of a thrombus results in serious vascular event such as acute myocardial infarction and stroke.

Arterial walls are thickened and stiffened with age (4, 5). This arterial remodeling process usually occurs at the level of elastic arteries, which is called “arteriosclerosis.” Loss of elastic fibers and increased fibrosis of arterial walls as a consequence of repetitive cyclic stress is the main pathophysiological feature of arteriosclerosis (4). Arteriosclerosis is the stiffening or hardening of the artery walls (= arterial stiffness), and it is different from the term “atherosclerosis,” which is the narrowing of the artery by the deposition of plaque (3). Atherosclerosis is a specific type of arteriosclerosis. Emerging evidence indicates that arterial stiffness is one of the earliest markers of functional and structural changes in arterial walls (5, 6). From clinical point of view, recognition, and measurement of arterial stiffness is important, because increased arterial stiffness is associated with worse cardiovascular outcomes, independent of traditional risk factors such as aging, hypertension, diabetes, dyslipidemia, obesity, and smoking (7–21). The most widely used measure of arterial stiffness is pulse wave velocity (PWV). As PWV is the measure of the speed of arterial pressure waves traveling along the aorta and large arteries, it is usually calculated by dividing distance with pressure wave transit time at the two points of recording arteries (5). The distance between the two sites along an arterial segment can be directly measured or estimated from the body height using regression model (22). The most commonly used method for estimating transit time is the foot-to-foot method: time difference between the feet of the two pulse waveforms (23). According to the two targeting arteries, various types of PWV measurements were determined such as carotid-femoral PWV (cfPWV) and brachial-ankle (baPWV). These two PWV are most widely used in clinical and research fields. In the measurement of aortic PWV, two arterial points are aortic root and descending thoracic aorta (24). Arterial pulse waveforms for PWV measurement can be obtained using applanation tonometry, echocardiography, and cardiac magnetic resonance imaging. Detailed information on specific method of each modality can be found elsewhere (5). PWV becomes faster in stiffened artery, and PWV value is elevated. The measurement of PWV is clinically valuable because it is simple, non-invasive, inexpensive, and reproducible (25).

Although there has been significant improvement in CVD prognosis by controlling risk factors such as hypertension, diabetes, and dyslipidemia, the burden of CVD is till substantial (1). It has generally been suggested that these classic risk factors cannot fully explain the increasing burden of CVD, and that more than 50% of patients with CVD do not have any of these risk factors (26, 27). In addition, many patients suffering from fatal CVD such as sudden cardiac death, myocardial infarction, or stroke do not have prior symptoms or warning signs (28, 29). Therefore, it is very important to detect subclinical atherosclerosis in early stages, and to identify individuals who are at high risk for future CVD such as angina, myocardial infarction, and stroke. Recently, special attention has been focused on PWV as a simple and reliable non-invasive tool to improve detection and risk stratification for CVD. This article will review the role of PWV in atherosclerosis and CVD.

## PWV in Coronary Artery Atherosclerosis

Recently, many studies have revealed the association between PWV and coronary atherosclerosis using cfPWV (17, 30–35) or baPWV (36–46). In symptomatic patients, PWV was associated with the presence and severity of coronary artery disease (CAD) assed by invasive coronary angiography (ICA) (17, 31, 32, 34, 36, 37, 40, 45). It was reported that there was a strong positive correlation between CAD severity and cfPWV (*r* = 0.838, *P* = 0.001) in 103 patients undergoing ICA (32). Hofmann et al. also used cfPWV as a measure of arterial stiffness in 155 patients undergoing ICA, and reported a positive association of elevated cfPWV with the presence and severity of CAD (34). Kim et al. investigated 501 patients with stable angina showed that baPWV is significantly associated with the presence and severity of CAD on ICA (40). In accordance with these findings, Xiong et al. measured baPWV in 321 symptomatic patients undergoing ICA, and demonstrated a significant correlation between baPWV and CAD severity assessed by SYNergy between percutaneous coronary intervention with TAXus and cardiac surgery (SYNTAX) score (45). Chung et al. reported the same finding of the positive correlation between baPWV and SYNTAX score in a study of 703 patients undergoing ICA (37). In asymptomatic diabetic population, both cfPWV and baPWV was associated with the presence and progression of coronary stenosis or plaque on computed tomography coronary angiography (CCTA) (33, 38). Our group investigated 470 patients with chest discomfort, and showed that baPWV has positive correlations with CAD extent and severity evaluated CCTA (39). Nam et al. evaluated 615 asymptomatic individuals, and demonstrated that elevated baPWV was an independent predictor of the presence of obstructive CAD detected by CCTA (42). Similarly, other studies enrolling healthy individuals consistently showed the positive association between PWV and coronary atherosclerosis assessed by luminal stenosis or coronary artery calcium (CAC) score on CCTA (35, 41, 43, 44). There have been a few longitudinal studies assessing the impact of baseline PWV on the development or progression of coronary atherosclerosis. Lee et al. investigated 1,124 subjects undergoing general health examination annually, and showed that baseline higher baPWV was significantly correlated with the progression of CAC during 2.7 year of follow-up (41). In a more recent study of 45 patients with type 2 diabetes, baseline cfPWV was associated with high-risk subtype of coronary plaque volume on CCTA measured after 5-years follow-up, independently of age, sex, diabetes, and blood pressure (33).

Summary of recent studies showing the association between PWV and coronary atherosclerosis is demonstrated in Table 1.

**Table 1:** Summary of recent studies showing the association between pulse wave velocity and coronary atherosclerosis.

**Source**	**Number of patients**	**Population**	**Mean age (years)**	**Design**	**PWV**	**Summary of findings**
Kim et al. (38)	83	Type 2 diabetes	64	Cross-sectional	baPWV	The AUC of baPWV for coronary artery stenosis (>20%) on CCTA was 0.672
Funck et al. (33)	45	Type 2 diabetes	63	Longitudinal	cfPWV	Baseline cfPWV was associated with high-risk subtype of coronary plaque volume on CCTA measured after 5-yearf follow-up, independently of age, sex, diabetes, and blood pressure
Chiha et al. (31)	344	Suspected CAD undergoing invasive CAG	61	Cross-sectional	cfPWV	cfPWV correlated with the extent of CAD, as measured by the “Extent” score (*r* = 0.21, *P* = 0.001)
Lee et al. (41)	1,124	Undergoing health check-up	44	Longitudinal	baPWV	Baseline higher baPWV was significantly correlated with the progression of CAC during 2.7 year of follow-up
Vishnu et al. (44)	1,131	Community population (men)	45	Cross-sectional	baPWV	baPWV was associated with the presence of CAC
Torii et al. (43)	986	Community population	986	Cross-sectional	baPWV	Prevalence of CAC progressively increased with rising levels of baPWV.
Cainzos-Achirica et al. (46)	15,185	Undergoing health check-up	42	Cross-sectional	baPWV	The multivariable-adjusted odds ratios for CAC > 0 comparing baPWV quintiles 2–5 vs. quintile 1 were 1.06, 1.24, 1.39, and 1.60, respectively (*P* trend < 0.001)
Duman et al. (32)	103	Suspected CAD undergoing invasive CAG	55	Cross-sectional	cfPWV	A highly positive correlation was observed between CAD severity and PWV (*r* = 0.838, *P* = 0.001)
Braber et al. (35)	193	Sportsmen	55	Cross-sectional	cfPWV	Adding cfPWV to traditional risk factor models did not change the AUC from 0.78 to AUC 0.78 (*P* = 0.99) for prediction of CAD on CCTA
Kim et al. (39)	470	Suspected CAD undergoing CCTA	470	Cross-sectional	baPWV	baPWV showed significant correlation with segment stenosis score, segment involvement score, CAC, and the number of segment with non-calcified plaque, mixed plaque, and calcified plaque on CCTA, respectively
Hofmann et al. (34)	155	Undergoing coronary bypass surgery	67	Cross-sectional	cfPWV	cfPWV was strongly associated with the severity of the patients' CAD (*P* < 0.001)
Chung et al. (37)	703	Suspected CAD undergoing invasive CAG	73	Cross-sectional	baPWV	baPWV was significantly associated with the SYNTAX score (*R*^2^ = 0.525, *P* < 0.001)
Kim et al. (40)	501	Suspected CAD undergoing invasive CAG	59	Cross-sectional	baPWV	baPWV was significantly associated with modified Gensini stenosis score (*P* = 0.033) and vessel disease score (*P* < 0.001) even after controlling for potential confounders
Chae et al. (36)	651	Suspected CAD undergoing invasive CAG	58	Cross-sectional	baPWV	baPWV was associated with the presence of obstructive CAD but not with CAD extent
Bechlioulis et al. (30)	393	Suspected CAD undergoing invasive CAG	61	Cross-sectional	cfPWV	Increased cfPWV was associated with CAD in overweight and obese patients (body mass index ≥ 25kg/m^2^; waist circumference ≥ 94 cm in men and ≥ 80 cm in women; *P* < 0.05)
Xiong et al. (45)	321	Suspected CAD undergoing invasive CAG	65	Cross-sectional	baPWV	Multivariable analysis showed that baPWV was independently associated with the SYNTAX score (*P* < 0.001)
Nam et al. (42)	615	Undergoing health check-up	53	Cross-sectional	baPWV	baPWV was associated with obstructive CAD on CCTA. The optimal cut-off value for the detection of obstructive CAD was 1,426 cm/s

*PWV, pulse wave velocity; baPWV, brachial-ankle pulse wave velocity; AUC, area under curve; CCTA, coronary computed tomography angiography; cfPWV, carotid-femoral pulse wave velocity; CAD, coronary artery disease; CAG, coronary angiography; CAC, coronary artery calcium; SYNTAX, SYNergy between percutaneous coronary intervention with TAXus and cardiac surgery*.

## PWV in Cerebral Artery Atherosclerosis

Cerebral small vessel disease (SVD), such as cerebral microbleeds, white matter hyperintensity, or lacunar infarction, is commonly observed in older people, and it is recognized as major vascular contributor to stroke, cognitive impairment, and geriatric syndrome (47, 48). Previous studies have reported an association between cerebral SVD and baPWV in the general population (49, 50) and patients with acute ischemic stroke (51, 52), lacunar infarction (53) or CAD (54). Similar finding showing the association between increased PWV and cerebral SVD were reported in studies using cfPWV (55–57) and aortic PWV (58–60). It has also been suggested that PWV is associated with cognitive dysfunction and the longitudinal progression of cognitive decline in the elderly (61, 62).

PWV is also associated with large cerebral artery calcification, stenosis, or occlusion. The association between increased cfPWV and cerebral artery calcification or stenosis has been revealed in hypertensive subjects (63) and in patients with acute ischemic stroke (64). A case-control study performed in Japan has indicated that a higher baPWV, in addition to traditional risk factors, is associated with a higher risk of cerebral infarction (65). In that study, baPWV ≥ 16 m/s had about three times higher risk of cerebral infarction compared to those with baPWV < 16 m/s. baPWV was significantly correlated with cerebral artery calcification or stenosis in patient with acute ischemic stroke (51, 66). However, there was also a negative report showing that a significant association between baPWV and ischemic stroke was abolished after controlling for potential confounders such as high blood pressure (BP) (67). The authors also showed that baPWV was not independently associated with the severity of white matter hyperintensity lesion or cerebral microbleeds (67).

Summary of recent studies showing the association between PWV and crebral atherosclerosis is demonstrated in Table 2.

**Table 2:** Summary of recent studies showing the association between pulse wave velocity and cerebral artery atherosclerosis.

**Source**	**Number of patients**	**Population**	**Mean age (years)**	**Design**	**PWV**	**Summary of findings**
Zhai et al. (50)	953	Community population	56	Cross-sectional	baPWV	Increased baPWV was associated with most of imaging markers of SVD, including dilated PVS in white matter, larger WMH volume, and marginally associated with strictly lobar CMB
Tabata et al. (54)	149	Coronary artery disease	71	Cross-sectional	baPWV	A multivariate analysis showed that baPWV were predictors of lacunar infarcts and CBM
Kim et al. (52)	1,282	Acute ischemic stroke or TIA	68	Cross-sectional	baPWV	On multivariate analysis, an increase in baPWV was associated with chronic lacunes, WMH, deep CMB, acute SVD, combined SVD score >1, and combined SVD score > 2
Rosano et al. (57)	273	Community population	83	Longitudinal	cfPWV	Higher cfPWV in 1997-1998 was associated with greater WMH volume in 2006-2008 within the left superior longitudinal fasciculus
King et al. (59)	1,270	Community population	51	Cross-sectional	aPWV	An increase in aortic PWV was related to an increase in subsequent WMH volume
Poels et al. (56)	1,460	Community population	58	Cross-sectional	cfPWV	Higher cfPWV was associated with larger white matter lesion volume but not with lacunar infarcts or microbleeds
Kim et al. (51)	801	Acute ischemic stroke	64	Cross-sectional	baPWV	Increased baPWV was associated with the presence of atherosclerosis (≥50% stenosis) in the intracranial cerebral artery, but not with atherosclerosis in the extracranial cerebral artery
Zhang et al. (63)	270	Hypertensive	61	Cross-sectional	cfPWV	cfPWV was independently associated with stenosis or calcification of intracranial artery
Kim et al. (53)	120	Lacunar infarction	64	Cross-sectional	baPWV	Patients with higher baPWV were more likely to have multiple lacunar infarcts and more severe white matter lesions
van Elderen et al. (60)	86	Type 1 diabetes	47	Cross-sectional	aPWV	Aortic PWV was independently associated with cerebral WMHs but not with cerebral microbleeds or lacunar infarcts
Ochi et al. (49)	443	Apparently healthy population	67	Cross-sectional	baPWV	OR of a high baPWV, defined as ≥1,500 cm/s, for the presence of CBM was 6.05 even after correction for confounding parameters, including age and hypertension
Brandts et al. (58)	50	Hypertensive	49	Cross-sectional	aPWV	Aortic PWV was statistically significantly associated with lacunar brain infarcts (OR = 1.8, *P* = 0.04), independent of age, sex, and hypertension duration, but not with WMH
Park et al. (66)	67	Acute ischemic stroke	65	Cross-sectional	baPWV	baPWV was significantly correlated with cerebral arterial calcification (*r* = 0.524, *P* < 0.001)
De Silva et al. (64)	268	Acute ischemic stroke	62	Cross-sectional	cfPWV	cfPWV was significantly higher in patients with significant stenosis of intracranial artery than those without
Choi et al. (67)	223	Stroke	66	Cross-sectional	baPWV	Multiple regression analysis revealed that the baPWV was not independently associated with increased risk of stroke, or the severity of WMH or CMB
Henskens et al. (55)	167	General population	52	Cross-sectional	cfPWV	A higher cfPWV was significantly associated with a greater volume of WMH and the presence of lacunar infarcts but not with CBM

*PWV, pulse wave velocity; baPWV, brachial-ankle pulse wave velocity; SVD, small vessel disease; PVS, perivascular space, WMH, white matter hyperintensity; CBMs, cerebral microbleed; TIA, transient ischemic attack; cfPWV: carotid-femoral pulse wave velocity; aPWV, aortic pulse wave velocity; OR, odds ratio*.

## PWV in Carotid Artery Atherosclerosis

Carotid intima-media thickness (IMT) is a marker of atherosclerosis, and is associated with CAD and stroke (68, 69). There was a positive linear correlation between carotid IMT and cfPWV in general population (70–73), hypertensive (74), and diabetic patients (72). Kubozono et al. first reported that high baPWV was a strong predictor of increased carotid IMT (≥1.0 mm) in 1,583 Japanese male subjects undergoing routine health check-up (75). Following cross-sectional studies have also showed the association between baPWV and increased carotid IMT or carotid plaque formation in general population (76), and patients with type 2 diabetes (77), end-stage renal disease (78), and cerebral artery thrombosis (79). Other PWV of other arterial tree segments including carotid-cerebral PWV (80), aorto-popliteal PWV (81), and heart-carotid PWV (82) also showed significant association with carotid atherosclerosis. A recently published longitudinal study showed that baPWV was independently associated with the risk of carotid plaque formation (83). As similar findings, baseline cfPWV was independently associated with an increase in carotid IMT during the 4-year follow-up (84). However, another study involving patients with acute ischemic stroke, revealed that baPWV was associated with the burden of intracranial atherosclerosis but not with extracranial carotid atherosclerosis (51). Lu et al. demonstrated that, only cfPWV, but not baPWV showed significant association with carotid IMT in general population (71).

Summary of recent studies showing the association between PWV and carotid atherosclerosis is demonstrated in Table 3.

**Table 3:** Summary of recent studies showing the association between pulse wave velocity and carotid artery atherosclerosis.

**Source**	**Number of patients**	**Population**	**Mean age (years)**	**Design**	**PWV**	**Summary of findings**
Fu et al. (80)	81	Acute ischemic stroke	63	Cross-sectional	ccPWV	ccPWV was independently associated with atherosclerosis between common carotid artery and middle cerebral artery
Yang et al. (83)	738	General population	52	Longitudinal	baPWV	Compared with baseline baPWV < 1,400 cm/s group, baPWV ≥ 1,400 cm/s group was significantly associated with the incidence of new carotid plaque formation even after adjusting for common risk factors
Sumbul et al. (74)	312	Hypertension	55	Cross-sectional	cfPWV	0.1 mm increase of carotid IMT was associated with increased cfPWV by 50%
Zhao et al. (84)	1,284	Hypertension	66	Longitudinal	cfPWV	Baseline cfPWV was independently associated with an increase in IMT of ≥1.5 *z*-scores during the 4-year follow-up
Lu et al. (71)	1,599	General population	73	Cross-sectional	cfPWV and baPWV	Only cfPWV, but not baPWV, showed significant association with carotid IMT
Kubozono et al. (75)	1.583	General population	56	Cross-sectional	baPWV	Carotid atherosclerosis (IMT ≥ 1.0 mm) was significantly associated with high baPWV
Joo et al. (76)	773	General population	55	Cross-sectional	baPWV	Subjects with higher baPWV was associated with higher prevalence of carotid artery plaque
Li et al. (82)	67	Hypertension	54	Cross-sectional	hcPWV	hcPWV was positively associated with carotid IMT
Koivistoinen et al. (81)	1,754	General population	30–45	Cross-sectional	apPWV	baPWV was independently associated with carotid IMT in older adults (β = 1.233, *P* = 0.019) but not in young adults
Shen et al. (70)	103	Elderly	69	Cross-sectional	cfPWV	cfPWV was significantly correlated with IMT (*r* = 0.322, *P* = 0.031), but not severity of carotid stenosis (*r* = 0.157, *P* = 0.313)
Kim et al. (51)	801	Acute ischemic stroke	64	Cross-sectional	baPWV	Increased baPWV was associated with the presence of atherosclerosis (≥ 50% stenosis) in the intracranial cerebral artery, but not with atherosclerosis in the extracranial cerebral artery
Tomonori et al. (79)	56	Cerebral thrombosis	65	Cross-sectional	baPWV	baPWV was associated with the existence of carotid plaque (*P* < 0.001)
Masugata et al. (77)	70	Type 2 diabetes	62	Cross-sectional	baPWV	baPWV correlated significantly with the carotid plaque score (*r* = 0.37, *P* = 0.001)
Munakata et al. (78)	68	End-stage renal disease	60	Cross-sectional	baPWV	baPWV was an independent risk factor for both plaque score (β = 0.006, *P* = 0.004) and maximum carotid IMR (β = 0.008, *P* = 0.04)
Zureik et al. (73)	564	General population	58	Cross-sectional	cfPWV	cfPWV was positively associated with carotid IMT (*r* = 0.39, *P* < 0.001) and lumen diameter (*r* = 0.42, *P* < 0.001) in sex-adjusted analysis
Taniwaki et al. (72)	271	Type 2 diabetes	51	Cross-sectional	cfPWV	There was a significant positive relationship between the carotid IMT and cfPWV (*r* = 0.482, *P* < 0.0001)

*PWV, pulse wave velocity; ccPWV, carotid-cerebral pulse wave velocity; baPWV, brachial-ankle pulse wave velocity; IMT, intima-media thickness; hcPWV, heart-carotid pulse wave velocity; apPWV, aorto-popliteal pulse wave velocity*.

## Predictive Value of PWV

The predictive value of PWV for the occurrence of CVD has been reported in the general population and patients with various clinical conditions. Mattace-Raso et al. investigated 2,835 community healthy subjects and showed that cfPWV is an independent predictor of coronary heart disease and stroke during 4 years of clinical follow-up (7). Another population study involving 1,678 community subjects over a median follow-up of 9.4 years, showed a similar finding that aortic PWV predicted composite cardiovascular outcomes beyond traditional risk factors (8). PWV also play a role as an independent predictor of future CVD in patients with hypertension (13, 16), diabetes (15), end-stage renal disease (17), stroke (14, 18), and CAD (19, 20).

There are several meta-analyses showing the prognostic value of PWV for the future cardiovascular events. A meta-analysis of 10 studies showed that increased cfPWV is a significant predictor of future CVD independent of Framingham risk factors (21). More recent meta-analysis of 19 studies have reported similar finding showing the association between cfPWV and CVD: 1 m/s increase of cfPWV was associated with 1.12-fold increase future CVD events (85). Findings based on individual participant meta-analysis of 17,636 subjects from 16 studies demonstrated that per 1-SD change in log_e_ cfPWV was an independent predictor for future CVD events by 1.45-fold (86). In regard to baPWV, a meta-analysis of 12 cohort studies indicated that an increase of 1 m/s of baPWV was associated with a 12% increase in the risk of CVD (9). More recently, meta-analysis of 14,673 Japanese participants without preexisting CVD showed that every 1-standard deviation (SD) increase of the baPWV was associated with a 1.19-fold increase in the risk of CVD during 6.4-year follow-up period (87).

## Mean and Cutoff Value of PWV

As PWV value can be affected by many clinical factors such as age and blood pressure, mean, or cutoff values of PWV are various among study population. However, PWV value are usually higher in patients with CVD than those without. cfPWV is higher in subjects with CVD (ranged 6 ~ 9 m/s) (34, 35, 88) than those without (ranged 8 ~ 13 m/s) (31, 34, 64, 88). In middle aged and elderly subjects, mean baPWV value has been reported ranged 15 ~ 18 m/s (42, 46, 75, 83), and 13 ~ 15 m/s (38, 40, 89) in with and without CVD or its risk factors, respectively. Several studies have indicated cutoff value of PWV in the prediction of atherosclerosis or cardiovascular events, which is summarized in Table 4. Current guidelines suggested that cfPWV > 10 m/s and baPWV > 18 m/s are indicative of individual's high risk in current guidelines in Europe (90) and Japan (25), respectively.

**Table 4:** Summary of recent studies showing cut-off value of PWV in the prediction of atherosclerosis or future cardiovascular events.

**Source**	**Number of patients**	**Population**	**Mean age (years)**	**Design**	**PWV**	**Summary of findings**
Kim et al. (38)	83	Type 2 diabetes	64	Cross-sectional	baPWV	Mean baPWV value of study population was 17.9 m/s. The optimal cutoff value of baPWV for the detection of coronary artery stenosis (≥20%) was 16.5 m/s with a sensitivity 68.9% and a specificity 63.2%
Yang et al. (83)	738	General population	52	Longitudinal	baPWV	Mean baPWV value of study population was 15.1 m/s. Compared with baseline baPWV < 14 m/s group, baPWV ≥ 14 m/s group was significantly associated with the incidence of new carotid plaque formation even after adjusting for common risk factors
Kubozono et al. (75)	1.583	General population	56	Cross-sectional	baPWV	Mean baPWV value of study population was 15.3 m/s. baPWV > 16.2 m/s was optimal cutoff value for detection of the presence of carotid atherosclerosis (carotid IMT ≥ 1 mm) (sensitivity 64% and specificity 71%)
Chiha et al. (31)	344	Suspected CAD undergoing invasive CAG	61	Cross-sectional	cfPWV	Mean cfPWV value of study population was 12.4 m/s. Patients with cfPWV ≥ 10 m/s was associated with higher coronary extent score than those with cfPWV < 10 m/s
Cainzos-Achirica et al. (46)	15,185	Undergoing health check-up	42	Cross-sectional	baPWV	Mean baPWV value of study population was 13.3 m/s. baPWV > 13.5 m/s had a sensitivity for CAC > 100 of 70% and a specificity of 59%. baPWV > 14.3 m/s had a sensitivity for CAC > 100 of 78% and a specificity of 51%
Kim et al. (39)	470	Suspected CAD undergoing CCTA	470	Cross-sectional	baPWV	Mean baPWV value of study population was 14.8 m/s. baPWV > 15.5 m/s was optimal cutoff value for detection of the presence and severity of obstructive CAD (≥ 50%) (sensitivity 56.6% and specificity 79.7%)
Lee et al. (89)	350	Suspected CAD undergoing myocardial SPECT	66	Longitudinal	baPWV	baPWV ≥ 17.9 m/s was independently associated with worse cardiovascular outcome
Braber et al. (35)	193	Sportsmen	55	Cross-sectional	cfPWV	Mean baPWV value of study population was 8.3 m/s. For the cfPWV > 8.3 m/s, the sensitivity to detect CAD was 43%, specificity 69%, positive predictive value 31% and negative predictive value was 79%
Chung et al. (37)	703	Suspected CAD undergoing invasive CAG	73	Cross-sectional	baPWV	Mean baPWV value of patients with CAD was 18.4 m/s. baPWV > 17.3 m/s had a sensitivity of 55.6% and specificity of 62.4% in predicting coronary stenosis
Kim et al. (40)	501	Suspected CAD undergoing invasive CAG	59	Cross-sectional	baPWV	Mean baPWV value of study population was 15.9 m/s. baPWV > 17 m/s was significantly associated with the presence and severity of obstructive CAD (≥ 50%)
Gasecki et al. (14)	134	Acute ischemic stroke	63	Longitudinal	cfPWV	Mean cfPWV value of study population was 8.3 m/s. cfPWV ≥ 9 m/s was associated with worse clinical outcome at hospital discharge with a specificity 61.5% and sensitivity 77.3%
Nam et al. (42)	615	Undergoing health check-up	53	Cross-sectional	baPWV	Mean baPWV value of patients with CAD was 14.3 m/s. The optimal cut-off value for the detection of obstructive CAD was 14.3 m/s, which had a sensitivity of 77% and a specificity of 63%

*PWV, pulse wave velocity; baPWV, brachial-ankle pulse wave velocity; CAD, coronary artery disease; CAG, coronary angiography; cfPWV, carotid-femoral pulse wave velocity; CAC, coronary artery calcium; SPECT, single-photon emission computed tomography*.

## Mechanisms Linking PWV and Atherosclerosis

**Figure 1:**
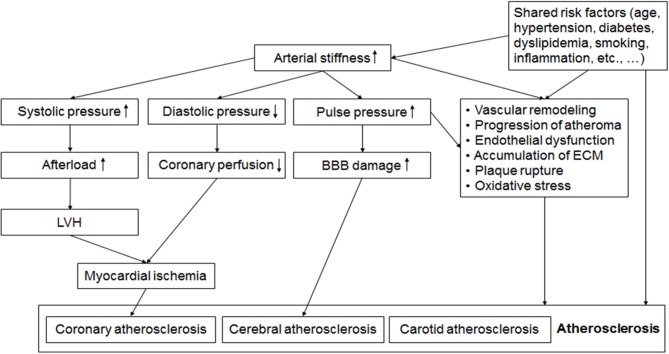
Possible mechanisms linking arterial stiffness and atherosclerosis. Alteration of pulsatile hemodynamics by increased arterial stiffness is associated with left ventricular hypertrophy (LVH), reduced coronary perfusion and the damage of blood brain barrier (BBB), which leads to coronary and cerebral atherosclerosis. Endothelial dysfunction, oxidative stress, vascular remodeling, accumulation of extracellular matrix (ECM), and shared common risk factors such as aging, hypertension, diabetes, and dyslipidemia are also factors linking increased arterial stiffness and the development and progression of atherosclerosis. BBB, blood brain barrier; ECM, extracellular matrix; LVH, left ventricular hypertrophy.

Mechanisms linking arterial stiffness and atherosclerosis have not been well-elucidated. However, several hypotheses could be suggested. Increased arterial stiffness leads to increased BP, and promote vascular remodeling (91). Also, increased luminal pressure and shear stress accelerates the formation of atheroma, and stimulates excessive collagen production and deposition in the arterial wall, leading to the progression of atherosclerosis (92). In addition, increased pulse pressure may be associated with the development of plaque and later its rupture (26). Indeed, both arterial stiffening and plaque formation depend partly on the same systemic pathophysiological process causing the accumulation of extracellular matrix in the arterial walls (3, 4, 93). Shared common risk factors such as hypertension, diabetes mellitus, and dyslipidemia may be another important mechanism linking PWV and atherosclerosis (5, 94). Mechanical aspect should be also considered in coronary atherosclerosis. Systolic BP increases and diastolic BP decreases in the stiffened artery (5, 95). Increased systolic BP and pulse pressure are hemodynamic burdens to the left ventricle leading to ventricular hypertrophy, and decreased diastolic BP is associated with reduced coronary perfusion (95, 96). Two well-known major risk factors for cerebral SVD are age and high BP (97), which are also major determinants of PWV. Increased pulse pressure by arterial stiffening may lead to endothelial dysfunction, and damages the microcirculation and blood-brain barrier, finally leading to both cerebral large artery atherosclerosis and SVD (95, 98, 99). Possible mechanisms linking arterial stiffness and atherosclerosis are demonstrated in Figure 1.

## Clinical Implications

Recognition of subclinical atherosclerotic vascular changes before clinical manifestation in an asymptomatic population is important for risk stratification and optimal management, which finally leads to the prevention of CVD (98, 100). Intensive modification of traditional risk factors has significantly reduced the development of CVD; however, the high residual prevalence of CVD requires further improvements in identification and risk stratification strategies. It has been suggested that the value of traditional risk factors such as hypertension, diabetes, dyslipidemia, and smoking for the identification of atherosclerotic burden in an asymptomatic population are limited (101). Also, several scoring strategies for CVD estimation are not sufficient to identify high risk subjects (102). In this purpose, several non-invasive tests such as carotid ultrasound, coronary computed tomography, and magnetic resonance imaging have been developed, and these tests are recommended for the evaluation of subclinical atherosclerosis especially in subjects with intermediate or uncertain risk (103). However, these imaging modalities are expensive, nephrotoxic, and hazardous in terms of radiation exposure and require specialized techniques or instruments. Arterial stiffening is one of the earliest manifestations of vascular damage, arteriosclerosis, and atherosclerosis. This functional and structural changes in arterial wall can be detected using PWV which a simple, non-invasive, simple and reliable measurement. As mentioned above, many cross-sectional and longitudinal studies have indicated that PWV can detect subclinical atherosclerosis and predict future CVD. PWV allows a greater convenience of screening subclinical atherosclerosis in the general population. Improved ability to identify high risk patients would lead to better risk stratification and more effective preventive therapy. Recent studies have also shown an additional role of PWV for the diagnosis and prediction of future cardiovascular events when combined with other tests (89, 104, 105). Additionally, PWV can be a target or monitoring tool for CVD risk-lowing therapeutic intervention (5, 95). Various pharmacological and non-pharmacological approaches able to reduce PWV (5) may offer potential advantages in the management of patients at high risk of CVD. However, further studies are required to confirm whether PWV reduction by this approach can directly prevent CVD.

## Comparisons Between cfPWV and baPWV

cfPWV measurement started in the 1960s (106), and thus, it has been most validated, and has a large amount of clinical data. cfPWV is a direct measurement, and it corresponds to the widely accepted propagative model of arterial system (107). Since aorta and its first branches are responsible for the most of the pathophysiological effects of arterial stiffness (107), cfPWV has been considered as the gold standard measurement of large artery stiffness (108). However, cfPWV measurement requires some skills, and carotid and femoral pulse acquisition are difficult especially in obese patients (107). In addition, palpation of carotid and femoral arteries causes patients' discomfort. By these reasons, the primary use of cfPWV remains in research settings, and it has not yet been implemented in clinical practice. In 2000, a more simplified method for the evaluation of arterial stiffness has been developed in Japan (109). Compared to cfPWV, baPWV is easier, less time-consuming, and less stressful for the patients, because baPWV value can be obtained by just wrapping pressure cuffs of upper arms and ankles. There have been many clinical data showing the usefulness of baPWV in the prediction of organ damage and cardiovascular outcomes in general population and patients with various medical conditions. However, there are several criticism on baPWV measurement (110): (1) baPWV includes large portion of peripheral muscular artery although muscular arteries may not be relevant for the CV risk assessment, (2) the height-based formula to estimate pulse transit distance for the calculation of baPWV is not validated, (3) the value of baPWV is usually higher than other PWV, because it overestimates arterial path lengths, and includes fast pulse wave traveling toward legs (22, 110), and (4) baPWV is underestimated in subjects with arterial stenosis of extremities, aortic aneurysm, or aortic stenosis, otherwise, it is overestimated in subjects with aortic regurgitation (111). Therefore, application of a more specific exclusion criteria is important in baPWV researches of cohorts with severe disease. It has been generally suggested that patients with ankle-brachial index < 0.9 should be excluded when using baPWV for any purpose (25, 111). Comparisons of strengths and limitations between cfPWV and baPWV are summarized in Table 5.

**Table 5:** Comparisons between cfPWV and baPWV (25, 110–112).

	**cfPWV**	**baPWV**
Strength	Includes only elastic arteries More abundant clinical data, and most validated Considered as gold standard a measure of arterial stiffness Widely used worldwide	Simple to measure Convenient to patients Useful in mass screening
Limitation	The measurement needs technical skill The measurement causes discomfort Less useful in mass screening	Includes both elastic and muscular arteries Invalid height-based formula to estimate arterial path length Inaccurate in patients with peripheral arterial stenosis or aortic disease Mainly used in Asian countries

## Limitations of PWV

Although information of PWV is useful in the early detection and risk prediction of cardiovascular disease, incorporation of PWV measurement in routine clinical practice has been barely performed, mainly due to several limitations of PWV. During the PWV measurement, distance between target points of arteries should be measured precisely because small inaccuracies may cause a greater error in the absolute value of PWV. Therefore, it is very important to recognize the differences in the methods used to assess path length, especially when comparing results between patient group and among different studies (107). In addition, the interpretation of PWV value should be cautious because many clinical factors affect the PWV value such as age, BP, and other cardiovascular risk factors (23). In particular, the most powerful confounders of PWV interpretation is BP. Increased BP augments the arterial wall tension and adds functional arterial stiffness. Therefore, BP effect should be controlled during the analysis using PWV. Although there have been few studies showing that several methods such as anti-hypertensive medications, exercise, statin, and smoking cessation improves arterial stiffness and decreases PWV value (5, 25), these results are from small-sized and non-randomized studies. Randomized control studies with large sample size on whether PWV-based management improves patients' outcome and the quality of daily clinical practice are required.

## Conclusions

PWV is well-correlated with the presence and extent of coronary, cerebral, and carotid atherosclerosis. More importantly, PWV had a predictive value in CVD beyond traditional risk factors in the general population and patients with various diseases. Considering its non-invasiveness, simplicity and abundant clinical data, measurement of PWV may be a useful tool to select subjects at a high risk of developing atherosclerosis especially in mass screening.

## Author Contributions

H-LK participate in drafting the manuscript, and S-HK participate in revising the manuscript.

### Conflict of Interest Statement

The authors declare that the research was conducted in the absence of any commercial or financial relationships that could be construed as a potential conflict of interest.
